# Retractile Testis in the Pediatric Population: Clinical Outcomes, Complications, and Management Implications

**DOI:** 10.3390/jcm15155846

**Published:** 2026-07-27

**Authors:** Maria Florou, Triantafyllia Koletsa, Sophia Tsokkou, Georgia Raptou, Ioannis Spyridakis, Christos Kaselas

**Affiliations:** 1 Second Department of Pediatric Surgery, “Papageorgiou” General Hospital, Aristotle University of Thessaloniki, 564 29 Thessaloniki, Greece; 2 Department of Pathology, School of Medicine, Aristotle University of Thessaloniki, 541 24 Thessaloniki, Greece; tkoletsa@auth.gr (T.K.); graptou@auth.gr (G.R.)

**Keywords:** retractile testis, acquired cryptorchidism, orchiopexy, fertility, torsion, testicular atrophy

## Abstract

**Background/Objectives:** Retractile testis has traditionally been considered a benign physiological variant, managed conservatively with observation until puberty. However, recent evidence suggests that retractility may predispose individuals to acquired cryptorchidism and complications such as torsion, testicular atrophy, and impaired fertility. This review aims to synthesize the current literature on the clinical features, natural history, complications, and management of retractile testes, and to evaluate whether conservative management remains appropriate. **Methods**: A structured narrative review was performed. PubMed (MEDLINE), Embase, Scopus, and the Cochrane Library were searched from database inception to 15 December 2025, and screening was reported using a PRISMA-style flow diagram. To our knowledge, this is the first review focusing exclusively on the retractile testis in the pediatric population, with a primary emphasis on management and treatment. **Results**: Prevalence among school-aged boys ranges from 1.2% to 3.9%. Reported progression rates to acquired cryptorchidism and orchiopexy vary widely, from 2% to 50%, with risk factors including inelastic spermatic cord, ipsilateral hernia, and younger age at presentation. Histological and sonographic studies demonstrate reduced testicular volume, seminiferous tubule degeneration, and impaired spermatogenesis, paralleling congenital cryptorchidism. Short-term complications include acute torsion, with abnormal gubernacular attachment and patent processus vaginalis frequently observed intraoperatively. Evidence for malignancy risk remains inconclusive. **Conclusions**: The available evidence, drawn largely from retrospective and observational studies, suggests possible associations between retractility and short- and long-term outcomes such as acute torsion, testicular atrophy, and impaired fertility, whereas an independent link with testicular malignancy remains unproven. This evidence is not yet sufficient to establish causation, and current professional-society guidelines continue to favor surveillance for the true retractile testis, reserving orchiopexy for documented ascent or other defined indications. Pending prospective data, these findings support structured, guideline-consistent follow-up, so that boys in whom ascent or atrophy develops are identified promptly.

## 1. Introduction

### 1.1. Definition and Conceptual Framework

Retractile testes are defined as testes that have descended physiologically into the scrotum at the time of birth, and that subsequently migrate intermittently to a higher, suprascrotal position during childhood. The displaced testis may rest along the inguinal canal, in the upper scrotum, or against the anterior wall of the inguinal canal—the so-called Dennis Brown (superficial inguinal) pouch [1,2]. Critically, during periods of cremasteric relaxation, the testis descends spontaneously, or can be brought down manually without traction, and remains in a dependent scrotal position at least transiently before retracting again. This distinguishes retractility from true congenital undescended testis, in which the gonad cannot reach the scrotum since birth and neonatal period, and from the ascending (acquired undescended) testis, in which a previously scrotal testis becomes progressively unable to remain in the scrotum [1].

The retraction is mediated by the cremasteric reflex, an arc carried by the genital branch of the genitofemoral nerve. Contraction of the cremaster muscle and consequent ascent of the testis can be triggered by a fall in ambient or skin temperature, stimulation of the inner thigh, abdominal wall contraction during play or examination, or autonomic arousal in response to fear or stress. The reflex is most active between the 2nd and 12th year of life, peaking around the 5th to 6th year, and gradually diminishes through puberty as testicular weight increases and the cremasteric muscle becomes proportionally less effective [3,4]. Because of this developmental trajectory, a retractile testis was historically considered a self-limiting phenomenon expected to resolve spontaneously by adolescence [3,5,6].

The classical management paradigm therefore consisted of reassurance and periodic re-examination until pubertal quiescence of the reflex, with surgery reserved for clear progression to a fixed undescended position. This watchful-waiting approach has, however, been progressively challenged. Concerns include uncertainty regarding the proportion of boys who ultimately require orchiopexy before adolescence, limited long-term andrologic data, and the inability to quantify the cumulative duration of extrascrotal residence and the consequent thermal exposure of germinal epithelium to temperatures 2–4 °C above the optimal scrotal milieu [3,5,7].

### 1.2. Embryology and Pathophysiology of Testicular Descent and Retractility

A nuanced understanding of testicular descent is essential to interpret retractility. Testicular descent occurs in two morphological and hormonally distinct phases. The first or transabdominal phase, completed by approximately 15 weeks of gestation, depends on insulin-like 3 (INSL3) acting through the LGR8/RXFP2 receptor to swell and shorten the gubernaculum, anchoring the testis to the inguinal region. The second or inguinoscrotal phase, occurring between roughly 25 and 35 weeks of gestation, is androgen-dependent and mediated indirectly via the genitofemoral nerve and calcitonin gene-related peptide (CGRP), which guides gubernacular migration toward the scrotum [8].

The cremaster muscle and fascia, derived from the internal oblique and transversus abdominis musculature, envelop the spermatic cord and testis and remain capable of vigorous contraction throughout childhood. In the retractile testis, the descent process has been completed normally, but a hyperactive or disproportionately strong cremasteric reflex repeatedly displaces the gonad superiorly. Failure of postnatal elongation of the spermatic cord relative to body growth, partial persistence of cranial gubernacular fibers, and a relatively redundant or short cord have all been implicated as contributing anatomic substrates [6,8].

When the testis spends prolonged intervals outside the scrotum, the germinal epithelium is exposed to core body temperature instead of the cooler scrotal environment maintained by the pampiniform plexus and dartos. Even modest, sustained thermal elevation accelerates germ cell apoptosis, impairs the transformation of gonocytes into adult dark spermatogonia, and disrupts Sertoli cell maturation [9]. These mechanisms provide a plausible biological rationale by which long-standing retractility, although by definition intermittent, may produce histologic and functional changes resembling those seen in congenital cryptorchidism.

### 1.3. Objective

The objective of the present review is to synthesize current evidence on the clinical features, diagnosis, natural history, complications, and management of the retractile testis—a common but increasingly contested clinical entity in school-aged boys—and to critically assess whether the traditional conservative posture remains justified in light of accumulating data on adverse short- and long-term outcomes.

## 2. Methodology

**Figure 1:**
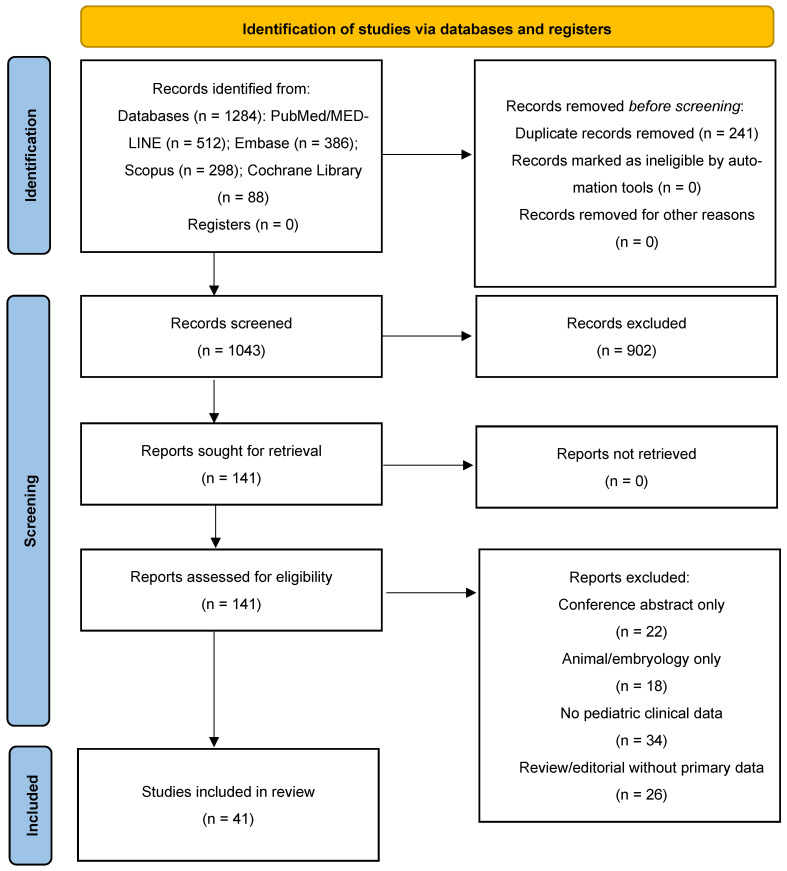
PRISMA-style flow diagram of study identification, screening, and inclusion. Records were identified by searching PubMed/MEDLINE, Embase, Scopus, and the Cochrane Library from database inception to 15 December 2025, supplemented by hand-searching of reference lists. After removal of duplicates, records were screened by title and abstract and then assessed by full text against the eligibility criteria, yielding the studies included in the narrative synthesis.

A comprehensive narrative literature search was conducted across PubMed (MEDLINE), Embase, Scopus, and the Cochrane Library from database inception to 15 December 2025 with no lower date limit applied. Search terms included “retractile testis,” “acquired cryptorchidism,” “ascending testis,” “orchiopexy,” “testicular torsion,” “testicular atrophy,” and “fertility,” combined with Boolean operators and Medical Subject Heading (MeSH) terms to refine results. The reference lists of pivotal articles, including the most-cited reviews and recent operative series, were hand-screened to identify additional studies not captured by the database queries. Records were screened by title and abstract and then by full text against the eligibility criteria below; the selection process is summarized in a PRISMA-style flow diagram (Figure 1). Eligible publications included peer-reviewed articles in English or Greek, published up to 2025, reporting on clinical features, case series, original observational studies, or meta-analyses. Conference abstracts without full texts, animal studies, and purely embryological or anatomical works lacking clinical correlation were excluded. Owing to the heterogeneity of definitions, diagnostic criteria, and follow-up durations across the included studies, a formal quantitative meta-analysis was not undertaken; results are presented as a structured narrative synthesis.

Because the retractile testis literature is dominated by small, retrospective, single-center cohorts, case series, and older historical reports with heterogeneous definitions and follow-up, we did not apply a formal quantitative risk-of-bias instrument; instead, the design and principal methodological limitations of each source (retrospective ascertainment, selection and referral bias, absence of control groups, and small sample sizes) are noted narratively when the corresponding evidence is discussed. This approach was chosen deliberately: the available body of evidence does not support the pooled effect estimates or graded causal inferences that a systematic review or meta-analysis would imply. Accordingly, the present work is framed as a narrative review, and its conclusions are intended to be descriptive and hypothesis-generating rather than prescriptive.

To our knowledge, this is one of the very first reviews dedicated exclusively to the retractile testis in the pediatric population; previous reviews have addressed retractility only as a subsection within broader treatments of undescended or ascending testis. The central question of this review is how the retractile testis should be managed, and its primary focus is therefore on management and treatment, with the clinical features, natural history, and complications presented insofar as they inform management decisions.

## 3. Findings

### 3.1. Prevalence, Clinical Presentation and Diagnosis

Retractile testes are most commonly identified in preschool and school-aged boys, although they may first be recognized after the third year of life as the cremasteric reflex becomes increasingly brisk. Recent large-scale epidemiological studies report prevalences ranging from approximately 1.2% to 3.9% among school-aged boys, with bilateral involvement in roughly one-third of affected children [10,11]. Diagnosis remains predominantly clinical, based on a focused personal history and a meticulous physical examination [8,10,11].

The perinatal history typically describes normally descended testes documented at birth and during early well-child evaluations. As the child grows, parents, primary-care clinicians, or school physicians often note that one or both testes intermittently disappear from the scrotum, particularly during bathing, urination, stress or play, and reappear during sleep or in a warm bath. A directed history should ascertain the position of the testis in neonatal period, the age at first observation, laterality, frequency, provocative factors, and whether the testis has ever been seen or felt within the scrotum.

Examination should be performed in a warm, calm environment with the child in a relaxed frog-leg position; if necessary, the examination can be repeated with the child standing or seated cross-legged (“tailor” position) to overcome anxiety-induced cremasteric activation. The clinician begins with inspection of the inguinoscrotal region, noting symmetry, scrotal hypoplasia, and any inguinal bulge. The cremasteric reflex is elicited by gentle stimulation of the inner thigh and graded for vigor and laterality. On palpation, the testis is typically located outside the hemiscrotum—along the inguinal canal, in the upper third of the scrotum, or in the Dennis Browne pouch. Critical examination steps include estimation of testicular size, consistency, and tenderness; assessment of the spermatic cord for elasticity or palpable indurated bands; gentle traction to bring the testis into the most dependent portion of the scrotum; and observation of whether the testis remains in the scrotum without immediate retraction once released [3,5,8].

Although diagnosis is clinical, scrotal high-frequency ultrasonography may be useful adjunctively to confirm intrascrotal position, to compare testicular volumes, to detect a patent processus vaginalis or communicating hydrocele, and to exclude an inguinal hernia. Hormonal assessment is generally not indicated in unilateral cases but may be considered in bilateral retractility associated with virilization or other endocrine concerns. Importantly, accurate differentiation from the ascending testis, the gliding testis, and the true undescended testis is essential, since these conditions follow distinct natural histories and have different management implications (Table 1).

### 3.2. Natural History

**Figure 2:**
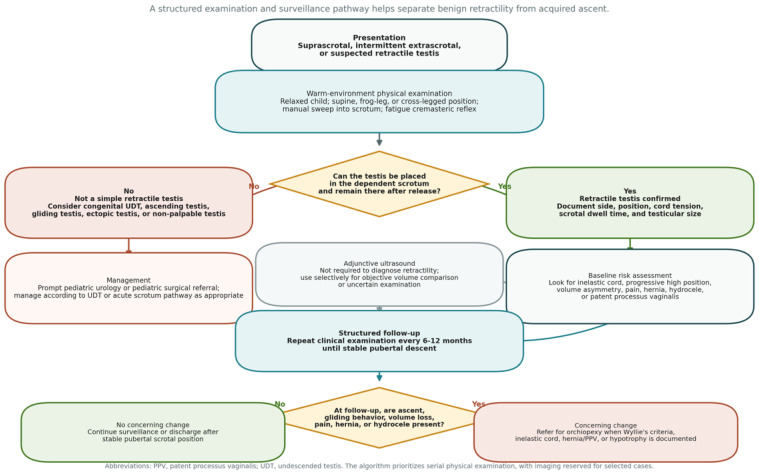
Proposed clinical evaluation and management algorithm for boys presenting with a retractile testis, integrating warm-environment examination, structured 6- to 12-month re-evaluation, sonographic comparison of testicular volumes, and individualized indications for orchiopexy when Wyllie’s criteria, an inelastic cord, ipsilateral hernia/patent processus vaginalis, or progressive ipsilateral hypotrophy are documented.

The natural history of the retractile testis is heterogeneous and remains incompletely characterized. In a substantial proportion of boys, the cremasteric reflex attenuates with age and the testis ultimately resides stably within the scrotum without intervention. In a clinically important minority, however, retractility evolves into acquired (ascending) cryptorchidism, in which the testis becomes progressively unable to be maintained in a dependent scrotal position because of a relatively short or inelastic spermatic cord, persistent fibrous remnants of the processus vaginalis, or abnormal gubernacular attachments [6,8,12,13]. Distinguishing these two trajectories prospectively at the index visit is currently not possible. This uncertainty is consistent with the periodic clinical re-examination recommended by current guidelines rather than discharge after a single reassuring encounter (Figure 2).

### 3.3. Management and Indications for Orchiopexy

#### Current Professional-Society Guideline Recommendations

Before considering how emerging evidence might modify practice, it is useful to summarize the current guideline landscape, as recommendations from the major professional societies are broadly concordant. The American Urological Association (AUA) defines a retractile testis as one that is initially extrascrotal or moves easily out of the scrotum, often with a vigorous cremasteric reflex, but that can be replaced in a stable, dependent scrotal position without tension and remain there at least temporarily; it advises assessing testicular position at least annually to monitor for secondary ascent and does not recommend surgery for a true retractile testis [14]. The European Association of Urology/European Society for Paediatric Urology (EAU/ESPU) similarly describe a testis that has completed descent but is found suprascrotally due to an overactive cremasteric reflex, recommend yearly controls until puberty in view of a secondary-ascent risk of up to about one-third, and reserve orchiopexy for documented ascent [15]. The Nordic consensus draws a careful operational distinction—a testis that remains in the upper or lower scrotum after cremasteric fatigue and release is “retractile” and is followed at least annually throughout childhood, whereas one that springs back immediately on release is classified as undescended and treated surgically; the Nordic statement additionally notes that treatment may be considered if there is progressive volume loss or histological deterioration [16]. The Canadian Urological Association (CUA) is the most explicit, stating that there is “no role for medical or surgical intervention” in a true retractile testis, recommending examination every 6 to 12 months and specialist referral only if ascent is documented [17]. Across these guidelines, the default posture is therefore surveillance rather than early surgery, hormonal therapy is not recommended, and routine imaging is not required; the principal surgical indication is documented secondary ascent to acquired cryptorchidism (Table 2). It should be emphasized that these recommendations are based largely on expert consensus rather than randomized evidence, and none formally applied the GRADE framework to the retractile testis.

Against this consensus, several strands of emerging evidence diverge from, or extend beyond, current guideline statements. First, reported ascent rates in some longitudinal cohorts (for example, approximately 21–32% at 2–5 years, and higher in boys under 6–7 years or with an inelastic cord) have led some authors to advocate more frequent, semi-annual surveillance in younger children rather than strictly annual review. Second, data on boys with bilateral ascending (acquired) testes suggest fertility potential impaired to nearly the same degree as in bilateral congenital cryptorchidism, prompting arguments for prompt surgery once ascent is confirmed and for closer andrologic attention in bilateral retractile cases. Third, torsion of a retractile testis, though uncommon, is reported in operative series and is not specifically addressed by guideline statements. These divergences do not overturn the guideline recommendation of surveillance for the true retractile testis, but they identify areas of genuine uncertainty and inform the individualized, structured follow-up advocated below [9,12,18,19,20,21].

Traditionally, the retractile testis has been managed conservatively through periodic clinical follow-up until puberty, on the assumption that spontaneous and definitive scrotal residence would ensue [7]. Within this framework, any meaningful clinical change identified during follow-up was considered an indication for surgical intervention and orchiopexy [22]. Three classical findings—commonly referred to as Wyllie’s criteria—have historically guided the decision to operate [23]:

1. A reduction of at least 20% in the volume or longitudinal diameter of the affected testis compared with the contralateral testis.
2. Tension of the spermatic cord that produces pain or visible cord shortening when the testis is brought into the hemiscrotum.
3. Immediate retraction of the testis out of the scrotum once it is released after manipulation [23].

Over time, this conservative strategy has been challenged on three principal grounds: (i) the lack of robust prospective data on the proportion of boys who ultimately undergo orchiopexy before puberty; (ii) limited long-term evidence regarding fertility, hormonal function, and malignancy risk; and (iii) the practical impossibility of accurately quantifying the cumulative duration of extrascrotal residence, and therefore the total exposure to temperatures 2–4 °C above the optimal scrotal milieu [6,9]. In addition, the contemporary literature has expanded the surgical decision matrix beyond Wyllie’s criteria to include intraoperative anatomic findings—patent processus vaginalis, ipsilateral inguinal hernia, abnormal gubernacular attachment—that have emerged as independent predictors of subsequent ascent and torsion risk [12,13,24,25].

Early work by Wyllie raised concern that up to 50% of boys with retractile testes might ultimately require orchiopexy [23]. Subsequent observational studies have reported strikingly variable progression rates. La Scala et al. (2004) followed 150 boys for a mean of 3.8 years and reported that 22.7% required surgery; risk was associated with spermatic cord tension and ipsilateral inguinal hernia, but not with family history of cryptorchidism or retractility [24]. Agarwal et al. (2006) studied 122 boys and found that 32% progressed to true cryptorchidism requiring orchiopexy before puberty, with higher risk in boys younger than 7 years and in those with a palpably inelastic cord [25]. Stec et al. (2007) reported a substantially lower surgical rate of 3.2% but again linked the need for surgery to a patent processus vaginalis and an inelastic cord [12]. Bae et al. (2012) found a 16.3% probability of needing surgery, higher in boys younger than 6.5 years, and noted that this risk was independent of the position of the contralateral testis [22]. The most recent large observational study from Japan reported that 30% of retractile testes ultimately progressed to secondary cryptorchidism necessitating orchiopexy [18]. Overall, reported progression rates range from approximately 2% to 50% (Table 3). A recent meta-analytic synthesis concluded that both the pathogenesis and the optimal management of retractile testes remain unsettled, framing the condition as a “grey zone” between physiological variant and pathological state [8].

### 3.4. Complications of Retractile Testis

**Figure 3:**
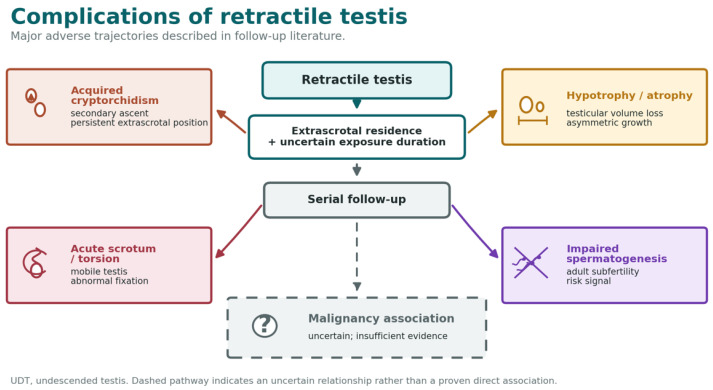
Short- and long-term complications of the retractile testis. Key. Solid arrows denote associations for which supporting clinical or histological evidence exists, albeit of variable strength: progression to acquired cryptorchidism [12,13,18,22,23,24,25]; testicular hypotrophy or atrophy [19,26,27]; acute scrotum due to torsion [13,28,29,30,31,32]; and impaired spermatogenesis with adult subfertility [20,21,33,34]. The dashed arrow denotes a hypothesized but unproven pathway—a direct, independent association between the retractile testis and testicular malignancy—for which current evidence is insufficient and limited to isolated reports [5,35]. Arrow style therefore reflects the strength of evidence rather than the magnitude of risk; UDT, undescended testis.

An expanding evidence base has prompted closer scrutiny of a purely expectant approach, citing wide variability in reported progression rates, sparse and heterogeneous long-term andrologic data, and the practical inability to determine how much cumulative time the testis spends outside the scrotum [6,9,19]. Reported complications fall into four broad categories: (i) acquired cryptorchidism with all of its sequelae; (ii) acute scrotum due to torsion; (iii) testicular hypotrophy and atrophy; and (iv) impaired spermatogenesis with subfertility in adulthood. The relationship to testicular malignancy remains uncertain (Figure 3, Table 4).

#### 3.4.1. Histologic and Andrologic Implications

The long-term consequences of untreated retractility were first recognized in adults with a documented childhood history of retractile testes who later presented with subfertility. In a retrospective study of men with preschool-onset retractility operated on during adolescence, testicular histology resembled that of congenital cryptorchidism, and only 21% had normal sperm counts [20]. Earlier reports from the 1980s had already suggested subfertility in this population [21].

In 1997, a detailed histologic study of testicular biopsies obtained from boys aged 1–9 years undergoing surgery for either retractile or congenital undescended testes demonstrated degenerative seminiferous tubule changes in retractile cases that were qualitatively akin to those of cryptorchidism [33]. A meta-analysis by Caroppo et al. summarized these findings, concluding that adults with a history of retractile testis exhibit reductions in sperm count and motility comparable to those of men with a history of congenital cryptorchidism [34]. Collectively, these data indicate that retractility can adversely affect spermatogenesis, particularly when surgical correction is delayed or omitted [20,21,33,34].

#### 3.4.2. Testicular Volume and Atrophy

Differences in testicular size and overt atrophy also appear to be shared by retractile and undescended testes, both clinically and on imaging. Ito et al. were among the first to demonstrate developmental disturbances in retractile testes resembling congenital cryptorchidism, including smaller testicular volumes, reduced seminiferous tubule counts, and spermatogonial alterations on biopsy [26]. Subsequent studies have confirmed smaller testicular volumes in boys with retractility relative both to the contralateral descended testis and to age-matched reference values [19,27]. These observations derive largely from small, cross-sectional studies and should be interpreted cautiously. Nonetheless, they support the role of serial ultrasonographic monitoring of testicular volume as a pragmatic surveillance tool; consistent with the Nordic consensus, progressive volume divergence may be one factor prompting consideration of orchiopexy.

#### 3.4.3. Acute Scrotum and Torsion

Short-term complications, particularly acute scrotum due to torsion, have been increasingly recognized in recent years. Case reports and small series describe boys with retractile testis presenting with acute scrotal pain and intraoperatively confirmed torsion [28,29]. It is generally accepted that an extrascrotal testis—whether congenital or acquired—has greater mobility and is more susceptible to torsion [29,30]. An elevated torsion risk has been described most robustly for frankly undescended testes; the extent to which it applies to the intermittently extrascrotal retractile testis is less well quantified and is supported mainly by case reports and small operative series. Mechanistically, abnormal gubernacular attachment, persistent processus vaginalis, an unfavorable testis-to-mesorchium width ratio, and heightened cremasteric activity have each been proposed as contributors to torsional susceptibility, and incompletely fixed testes subjected to vigorous cremasteric contractions may be more vulnerable to twisting [30,31,32]. A 2023 operative study reported abnormal gubernacular attachment in 75% and a patent processus vaginalis in 56% of retractile cases, mirroring findings in congenital cryptorchidism [13]. These anatomical observations are consistent with prior series and are compatible with a plausible, though not firmly quantified, torsion risk in this group [13,36,37,38].

#### 3.4.4. Malignancy Considerations

Evidence is currently insufficient to establish a direct, independent association between retractile testis and an increased risk of testicular malignancy. Only isolated reports describe patients in whom a retractile testis progressed to secondary cryptorchidism and later underwent malignant transformation [5,35]. Acquired cryptorchidism is itself a recognized—if quantitatively less well-defined—risk factor for malignancy, but a direct link between retractile testis per se and germ cell tumors remains unproven. Pragmatically, this argues for incorporating self-examination education and routine genital examination into long-term follow-up of boys with a documented history of retractility, particularly those who progressed to ascent or required orchiopexy.

### 3.5. Synthesis: Outcomes and Clinical Implications

Table 4 synthesizes the principal complications associated with the retractile testis, the strength of supporting evidence, and the clinical implications for surveillance and surgical decision-making. Because most of these associations rest on low-certainty evidence, they are best used to inform individualized, guideline-consistent surveillance rather than to mandate a uniform change in practice. Considered together, the data support structured follow-up in which surgery is considered when defined risk markers most importantly documented ascent are present, in keeping with current professional-society recommendations.

### 3.6. Counseling, Follow-Up, and Practical Recommendations

Children with a clinically diagnosed retractile testis should be entered into a structured follow-up program rather than discharged after a single reassuring encounter. A pragmatic schedule includes review every 6 to 12 months until puberty, with each visit comprising a warm-environment examination, documentation of testicular position and elasticity of the spermatic cord, and—when feasible—measurement of testicular volume by orchidometer or scrotal ultrasonography. Caregivers should be counseled regarding the rationale for surveillance, the warning signs of acute scrotum (sudden severe pain, scrotal swelling, nausea, and tenderness) that require urgent evaluation, and the indications that may shift management toward orchiopexy. Adolescents should receive instruction in monthly testicular self-examination, particularly if they have undergone orchiopexy or experienced documented ascent.

From a surgical standpoint, contemporary practice increasingly favors orchiopexy when one or more of the following are documented during surveillance: fulfillment of one or more Wyllie criteria; a palpably inelastic spermatic cord; an associated ipsilateral inguinal hernia, communicating hydrocele, or patent processus vaginalis; progressive ipsilateral testicular hypotrophy; bilateral retractility with concerning andrologic markers; or an episode of acute scrotum [6,12,13,22,23,24,25]. Operative technique typically follows standard inguinal orchiopexy with high ligation of any patent processus vaginalis, careful dissection of the spermatic cord to maximize length, and dartos pouch fixation. Intraoperative findings—particularly anomalous gubernacular insertion and vasoepididymal disjunction—should be systematically documented because they appear to identify a subset of children at higher residual risk of complications [13,36,37,38].

### 3.7. Limitations of the Current Evidence Base

Despite an expanding literature, the evidence base on retractile testes remains substantially limited. Most available studies are small, retrospective, and single-center, and they employ heterogeneous diagnostic criteria, varying age cutoffs, and inconsistent follow-up durations. Reported progression rates therefore differ widely—often by an order of magnitude—across cohorts that nominally describe the same condition, complicating both clinical translation and quantitative synthesis. Long-term andrologic and oncologic data are particularly sparse; very few cohorts have followed boys diagnosed with retractility into adulthood with structured semen analysis or hormonal assessment. Most fertility data derive from selected adult populations attending infertility clinics, introducing referral bias. Standardized definitions, prospective multicenter registries, and harmonized outcome set that include andrologic, oncologic, and patient-reported outcomes are needed to advance the field.

### 3.8. Directions for Future Research

Several priorities for future investigation emerge from this synthesis. First, prospective multicenter cohorts with standardized diagnostic criteria—ideally separating retractile, gliding, and ascending testis at enrollment—are needed to define accurate progression rates and to identify reliable predictors of ascent. Second, longitudinal andrologic follow-up of these cohorts into adulthood, including semen analysis, reproductive hormone profiling, and time-to-conception data, would clarify the true fertility implications of childhood retractility. Third, randomized comparisons of structured surveillance versus early orchiopexy in defined risk strata (for example, boys aged less than 6 years with an inelastic cord or bilateral retractility) would directly address the central management uncertainty. Fourth, mechanistic studies integrating histologic, sonographic, and molecular markers may help to stratify risk at the index visit. Finally, the psychosocial dimensions of repeated genital examination and prolonged uncertainty deserve formal investigation, ideally through validated patient-reported outcome instruments [22,23,24].

In summary, the available literature suggests that retractile testis is best regarded as a contested clinical entity rather than a uniformly benign normal variant. Reported progression rates to acquired cryptorchidism vary widely across cohorts—from roughly 2% to 50%, with several studies clustering around 20–32%—which reflects heterogeneous definitions and follow-up as much as true biological variation [12,18,22,23,24,25]. The remaining outcomes described for the retractile testis—greater mobility and a possible increase in torsion risk, testicular hypotrophy and reduced volume, and long-term subfertility, with uncertain implications for malignancy—are supported chiefly by retrospective and observational data and cannot yet be regarded as established causal effects. Taken together, and in line with current guidelines, these observations support structured surveillance with individualized indications for surgery, rather than either undifferentiated reassurance or a blanket move toward earlier operation.

## 4. Conclusions

Interpreted in the context of contemporary evidence, the retractile testis appears to be a distinct clinical entity that shares multiple features with congenital cryptorchidism rather than a uniformly benign normal variant. Congenital cryptorchidism is a well-defined pathological condition with established short- and long-term complications, and the central question raised by the retractile testis literature is the extent to which the demonstrated similarities between retractile and cryptorchid testes translate into clinically meaningful outcomes—particularly acute torsion, testicular atrophy, subfertility, and possibly malignancy—and whether these parallels justify modifying the traditional conservative approach.

The recent literature has prompted re-examination of a purely expectant strategy that relies on a single reassuring encounter. Importantly, however, current professional-society guidelines—including those of the AUA, the EAU/ESPU, the Nordic consensus, and the CUA—continue to recommend surveillance rather than primary surgery for the true retractile testis, with orchiopexy reserved for documented secondary ascent (and, in some guidelines, progressive volume loss). These recommendations rest largely on expert consensus rather than randomized evidence. Consistently with this position, the practical message of the present review is that boys with retractile testes are best entered into a structured re-examination program—commonly annual or 6- to 12-monthly in younger children per the CUA—with documentation of testicular position, cord elasticity, and, where feasible, volume. Within this framework, orchiopexy is considered on an individualized basis when guideline-recognized indications emerge, most importantly documented ascent, and when locally relevant risk factors (such as an inelastic cord, ipsilateral hernia or patent processus vaginalis, or progressive ipsilateral hypotrophy) are present. Until robust prospective data—ideally from standardized multicenter cohorts and, where feasible, randomized comparisons—become available, retractile testis is best approached as a condition warranting active, guideline-consistent surveillance rather than either single-encounter reassurance or a routine shift toward earlier operation, mindful of the risks and costs of unnecessary surgery in the substantial proportion of boys who resolve spontaneously.

## Figures and Tables

**Table 1:** Differential diagnosis of suprascrotal testicular positions in childhood [1,2,3,5,8].

Entity	Position at Rest	Manual Scrotal Placement	Stays in Scrotum After Release	Typical Management
Retractile testis	Suprascrotal (inguinal canal, upper scrotum, Dennis Browne pouch); intermittently scrotal	Easily delivered without tension	Yes—remains scrotal at least temporarily	Observation with structured follow-up; surgery if criteria emerge
Gliding testis	High scrotal/external ring; rarely descends spontaneously	Can be brought down with gentle traction	No—immediately retracts on release	Generally orchiopexy
Ascending (acquired undescended) testis	Previously scrotal; now persistently extrascrotal	May or may not be deliverable; cord short/inelastic	No	Orchiopexy when ascent is documented
Congenital undescended testis (palpable)	Inguinal canal or external ring from birth; never documented in scrotum	Limited descent against tension	No	Orchiopexy, ideally between 6 and 18 months
Congenital undescended testis (non-palpable)	Intra-abdominal, canalicular, or absent	Not palpable	Not applicable	Diagnostic laparoscopy ± staged orchiopexy/orchiectomy

**Table 2:** Summary of current professional-society guideline recommendations for the retractile testis [14,15,16,17].

Society (Year)	Definition of Retractile Testis	Follow-Up Interval	Surgical Indication/Posture
AUA (2014/2018)	Extrascrotal or moves easily out; replaceable in a stable dependent scrotal position without tension, remains at least temporarily	At least annually until puberty	Surgery on documented secondary ascent
EAU/ESPU (2025)	Completed descent but found suprascrotal due to overactive cremasteric reflex; can be manipulated down and remains at least temporarily	Yearly controls until puberty	Orchiopexy only on documented ascent
Nordic consensus (2007)	Remains in upper or lower scrotum after cremasteric fatigue and release (springs back immediately = undescended)	At least once a year throughout childhood	Orchiopexy on ascent; may be considered for progressive volume loss
CUA-PUC (2017)	Intermittently migrates via a brisk cremasteric reflex; manipulated to normal position and remains after release	Every 6 to 12 months	Surveillance; orchiopexy only on ascent

**Table 3:** Summary of major studies reporting progression to orchiopexy in boys with retractile testes [8].

Study	Sample Size	Rate of Surgery/Progression	Key Risk Factors
Wyllie 1984 [23]	91	Up to 50%	Not specified
La Scala et al. 2004 [24]	150	22.7%	Inelastic spermatic cord; ipsilateral inguinal hernia
Agarwal et al. 2006 [25]	122	32%	Age < 7 years; inelastic cord
Stec et al. 2007 [12]	172	3.2%	Patent processus vaginalis; inelastic cord
Bae et al. 2012 [22]	108	16.3%	Age < 6.5 years
Hori et al. 2021 [18]	145	30%	Not specified

**Table 4:** Summary of outcomes and complications of retractile testis with clinical implications.

Outcome/Complication	Mechanism/Contributing Factors	Strength of Evidence	Clinical Implication
Progression to acquired cryptorchidism	Inelastic spermatic cord; persistent processus vaginalis; abnormal gubernaculum; younger age at presentation	Multiple observational cohorts; range 2–50% [12,18,22,23,24,25]	Structured 6–12-month re-examination; orchiopexy when ascent documented
Testicular hypotrophy/atrophy	Chronic thermal exposure; impaired Leydig and Sertoli cell function	Multiple sonographic and histologic studies [19,26,27]	Serial ultrasound; volume divergence ≥ 20% as surgical trigger
Impaired spermatogenesis/subfertility	Germ cell apoptosis; reduced spermatogonial counts; delayed gonocyte transformation	Histologic and andrologic studies [20,21,33,34]	Counsel families regarding fertility risk; consider earlier orchiopexy in bilateral or high-risk cases
Acute scrotum/intravaginal torsion	Increased mobility; abnormal gubernacular attachment; vigorous cremasteric reflex	Case series and operative cohorts [13,28,29,30,31,32]	Patient and parent education; low threshold for emergency evaluation of acute scrotal pain
Testicular malignancy	Possible indirect risk via progression to acquired cryptorchidism	Isolated reports; no independent association established [5,35]	Long-term follow-up; teach testicular self-examination from adolescence
Psychosocial impact	Recurrent examinations; uncertainty regarding need for surgery	Limited [13,28,29,30,31,32]	Clear communication; written counseling material; involve child where developmentally appropriate

## Data Availability

No new data were created or analyzed in this study. Data sharing is not applicable to this article.
